# Supplementary material for: Case Study Using Recommended Reference Genes Actin and 18S for Reverse-Transcription Quantitative Real-Time PCR Analysis in Myzus persicae
Source: PLoS One. 2021 Oct 20;16(10):e0258201. doi: 10.1371/journal.pone.0258201 (PMC8528319; doi:10.1371/journal.pone.0258201)
Supplement: Attachment — Submitted filename: Response to Reviewers.docx [file pone.0258201.s001.docx]

**Response to Reviewer 1**

**Reviewer #1: The manuscript written by Rahman et al. reported that actin but not 18s can be used to analyze gene expression at development stages of *Myzus persicae*. This is a basic work but is very important for studying molecular biology of *M. persicae*. Overall, the experiments were well designed and the manuscript was well written. I only have a few minor concerns:**
**Response:**
 We are very grateful to your comments on the manuscript. At the same time, we have thoroughly revised the manuscript in order to express the idea more clearly. We believe the manuscript has been greatly improved. Once again, thank you for the kind advice and acknowledgments.
**1. In page 4, line 3, five methods should be revised as** **one method and three software.**

**Response:**

**DONE.**

“…five methods…” was changed to “one method and three software”.
**2. In figure 2, 3 and 4, please put “Actin”, “18S”, “Actin and 18S” into each panel. This will make the figures clear.**

**Response:**

**DONE.**

“Actin”, “18S” and “Actin and 18S” were added into each panel.
**3. In page 9, line 18, you should give the full name when first used.**

**Response:**

**DONE.**

The full name was added for each gene when it was used firstly, as follow. “…..*ribosomal protein L* (*RPL*), *Tublin*, *GADPH*, *ribosomal protein S* (*RPS*), *18S*, *EF1A*, *TATA box binding protein* (*TATA*), *heat shock protein* (*HSP*) and *succinate dehydrogenase complex subunit A* (*SDHA*)”.
**4. In page 17, line 24, “under the same treatment of one insecticide”?**

**Response:**

**DONE.**

Page 14, lines 15-18, “among different concentrations…under the same treatment of one insecticide…” was changed to “…..among different starvation time…..under the same starvation time”. Page 14, lines 23-26, “among different concentrations…under the same treatment of one insecticide…” was changed to “among different developmental stages ….under the same developmental stage”.

**Response to Reviewer 2**

**Reviewer #2: This manuscript by Rahman et al. validated the stability of two reference genes Actin and 18S by analyzed the relative expression levels of four target genes. I have some questions for this research.**
**Response:**

We are very grateful to your comments on the manuscript. At the same time, we have thoroughly revised the manuscript in order to express the idea more clearly. We believe the manuscript has been greatly improved. Once again, thank you for the kind advice and acknowledgments.

**1. First, it is better to standardize the nomenclature associated with quantitative PCR to avoid confusion; for example, the abbreviation RT-PCR should be used for reverse transcription PCR, qPCR should be used for quantitative real-time PCR, and RT-qPCR should be used for reverse transcription-qPCR. However, in this study, the abbreviations relative to PCR is very confusing; there are four types of abbreviation, "real-time qRT-PCR", "real-time RT-qPCR", "RT-qPCR" and "qRT-PCR".**

**Response:**

**DONE.**

We checked this manuscript carefully and standardized the nomenclature associated with quantitative PCR to only one type, RT-qPCR.
**2. Second, since the purpose of this study is to validate the stability of two reference genes, it is necessary to show all the Ct values if possible.**
**Response:**

**DONE.**

The mean *Ct* and standard error values of of *Actin* and *18S* for all samples in this paper were shown in the section of “Expression of target genes normalized to Actin and 18S at different developmental stages of *M. persicae*”.

**3. Third, consider to rearrange Figure 2-4. It is hard for reader to compare the tendency of the relative expression levels of one target gene (normalized to Actin, 18S, or two-gene combination). I think it is better to put the three results of one same gene (normalized to Actin, 18S, or two-gene combination) into an independent line chart. Then combine the four graphs (four target genes that with the same treatment) together.**
**Response:**

**DONE.**

Thanks for your good suggestion. We reviewed Figures 2-4 according to the suggestions of Reviewer 1. We added “Actin”, “18S”, “Actin and 18S” into each panel.
**4. Fourth, it is not nice for reviewer to make comment when the line number of each page is independent.**
**Response:**

**DONE.**

We have numbered each line of this paper continuously.

**In addition, the followings should be considered in revising the manuscript.**
**5. Page 2,**

**Line 13: The initial of actin should be capitalized. Please check the full manuscript.**

**Response:**

**DONE.**
 We have checked carefully the full manuscript and corrected.
**6. Page 3, line 9: quantify gene expression.**

**Response:**

**DONE.**

“….quantity RNA expression…” was changed to “….quantity gene expression…”.
**7. Page 4,
Line1: the appropriate abbreviation of ribosomal protein L27 should be RPL27. And RPL32 should be italic.**

**Response:**

**DONE.**

“L27” was changed to “*RPL27*” and RPL32 was in italic type.
**Line 10-12: why you chose this four target genes has to be mentioned in introduction.**

**Response:**

**DONE.**

We have given the reasons as follow: CYP6CY3 and E4 are belong to metabolic enzymes, which have been studied widely and deeply and associated to insecticide resistance in *M. persicae* [1]. The over-expression of these two genes induced by insecticide treatment even as sublethal doses have been observed in many insects including *M. persicae* [20-22]. CYP6-1 is a member of cytochrome 450 monooxygenase family. VPS11 is involved in vesicle transport to vacuoles which plays an important role in segregation of intracellular molecules into distinct organelles [23]. However, both CYP6-1 and VPS11 are unfamiliar in *M. persicae*. The changes in the expression pattern of these two genes induced by the changes of both biotic and abiotic conditions would be unpredictable, which would give a relative comparison.
**Line 21: need format modification. Please check the full manuscript.**
**Response:**

**DONE.**

“….a 16 L:8D photoperiod…” was changed to “….a 16 Light: 8 Dark photoperiod…”. And We check the full manuscript carefully.
**8. Page 5,
Line 11: Adult aphids used in the study are female only, male only, or random. And how many days have the adults emerged.
Response:**

**DONE.**

This section was change to “Five different growing stages of *M. persicae* (green peach aphids), including 1st, 2nd, 3rd, and 4th instar nymphs and newly emerged adults, were collected as samples [20]. For each nymph sample, a total of 60 aphids were collected, and for each adult sample, total random 30 aphids were collected.”

**Line 24, 25: The cDNAs have been synthesized by reverse transcription PCR, hence, only qPCR here. It has to be clarified if the "qRT-PCR" here stands for "quantitative real-time PCR".**
**Response:**

**DONE.**

We checked this manuscript carefully and standardized the nomenclature associated with reverse transcription PCR and quantitative real-time PCR.
**9. Page 6,
Line 9: Pfaffl [6]**
**Response:**

**DONE.**

“Pfaffl^6^” was changed to “Pfaffl [6]”.

**Line 26-28: It is better to count the numbers to same decimal places.**
**Response:**

**DONE.**

The data was unified to the same decimal places.

**10. Page 7,
Line 3: The mortalities were too low and have no differences among three concentrations. It might be exposure to insecticides only for 24 h or the concentrations of these two insecticides might be not appropriate. Besides, the mortality data of control was not listed in Table 2.**

**Response:**

**DONE.**

Firstly, the mortality data of control group was listed in Table 2. Secondly, the population used in this study is a field population collected from Nanping, Fujiang Province in 2018 (Sial et al., 2020, [3]), which have developed a certain level of resistance to pesticides. Therefore, the concentrations of tested two insecticides used this study just induced sublethal effects. And the highly mortalities were not the aim of this study. The metabolic resistance mechanism related to the over-production of carboxylesterases *E4* and Cytochrome P450 monooxygenases *CYP6CY3* seem to confer low to moderate levels of resistance to pesticides in *Myzus persicae*, which have been documented in a lot of paper. In a word, the changes in the expression of metabolic enzymes induced by sublethal-doses of pesticides were detected in this paper.
**Line 10, 26: I noticed that the differences only occurred at one or two tested time point, and not all target genes show differences when normalized with Actin, 18S, or two-gene combination. It seems that the difference occur relative to the expression profile of different target gene or the deviation among samples. These results were not persuasive to evaluate the stability of the reference gene.**
**Response:**

**DONE.**

This section was changed to “As shown in Fig 3, the tendency of expression changes of the four target genes normalized to *Actin* was generally consistent with those normalized to *18S* and was initially inhibited and then promoted with changes in starvation time (Figs 3A and B). After starvation for 6h, the relative expression levels of four target genes were all inhibited significantly compared to those without starvation treatment (starvation for 0 hours) normalized both with *Actin* and *18S* as reference gene respectively, except for *E4* and *VSP11* normalized with *Actin*. The relative expression levels of four target genes were all increased significantly in *M. persicae* after starvation for 48h normalized both with *Actin* and *18S* as reference gene respectively. A comparison between the relative expression levels normalized with *Actin* and *18S* showed that significant differences occurred for all tested target genes, with *CYP6CY3* at 12 h and 48 h, *CYP6-1* at 6 h, *E4* at 12 h, and *VPS11* at 6 h and 12 h. As shown in Fig 3C, the expression fold normalized to the combination of *Actin* and *18S* showed no differences compared with those normalized to *18S* individually (Figs 3B and C).”
**11. Page 8,
Line 8: Define Ct. Besides, for example, 16.51±1.51 indicated mean** **±** **standard error or standard deviation.**
**Response:**

**DONE.**

The define “threshold cycle” was added for *Ct*. And “….the *Ct* values….” were changed to “mean threshold cycle (*Ct*) and standard error values” in lines 8 and 10.
**12. Page 9,
Line 18: Define RPS, TATA, HSP and SDHA.**
**Response:**

**DONE.**

The full name for RPS, TATA, HSP and SDHA were added.
**13. Page 10,
Line 12: The reference genes were ranked from the most stable to the least stable, but the "<" here was very confusing when lack of enough background. I think it is not appropriate to copy exactly the description in the reference.**

**Response:**

**DONE.**

It was changed to “>”.
