# Supplementary material for: Case Study Using Recommended Reference Genes Actin and 18S for Reverse-Transcription Quantitative Real-Time PCR Analysis in Myzus persicae
Source: PLoS One. 2021 Oct 20;16(10):e0258201. doi: 10.1371/journal.pone.0258201 (PMC8528319; doi:10.1371/journal.pone.0258201)
Supplement: Attachment — Submitted filename: Response to Reviewers.docx [file pone.0258201.s002.docx]

**Response to Reviewer 2**

**Reviewer #2: The followings should be still considered in revising the manuscript.**

**Response:**
 We are very grateful to your comments on the manuscript. At the same time, we have thoroughly revised the manuscript in order to express the idea more clearly. We believe the manuscript has been greatly improved. Once again, thank you for the kind advice and acknowledgments. **1. Line 169 and 173, add a space between number and "h".**

**Response:**

DONE. A space was added between number and “h”.  **2. Line 206, italicize the "E".**

**Response:**

DONE. The “E”was changed to “*E*”. **3. Line 207, there is no Fig 5 in this manuscript.**

**Response:**

DONE. The Fig 5 was submitted. **4. Line 277, delete the space between "-" and "q".**

DONE. The space was deleted between "-" and "q".
